# Phenylmethimazole Blocks dsRNA-Induced IRF3 Nuclear Translocation and Homodimerization

**DOI:** 10.3390/molecules171012365

**Published:** 2012-10-22

**Authors:** Maria C. Courreges, Noriko Kantake, Douglas J. Goetz, Frank L. Schwartz, Kelly D. McCall

**Affiliations:** 1 Department of Specialty Medicine, Ohio University, Athens, OH 45701, USA; 2 Interthyr Corporation, Athens, OH 45701, USA; 3 Biomedical Engineering Program, Ohio University Russ College of Engineering & Technology, Ohio University, Athens, OH 45701, USA; 4 Department of Chemical and Biomolecular Engineering, Ohio University Russ College of Engineering & Technology, Ohio University, Athens, OH 45701, USA; 5 Diabetes Institute, Ohio University College of Osteopathic Medicine, Ohio University, Athens, OH 45701, USA; 6 Department of Biological Sciences, Ohio University College of Arts & Sciences, Ohio University, Athens, OH 45701, USA; 7 Molecular & Cellular Biology Program, Ohio University College of Arts & Sciences, Ohio University, Athens, OH 45701, USA

**Keywords:** phenylmethimazole (C10), IRF3

## Abstract

Previous studies revealed that phenylmethimazole (C10) inhibits IRF3 signaling, preventing dsRNA-induction of type 1 interferon gene expression, production, and downstream signaling. In the present study, we investigated the molecular basis for C10 inhibition of dsRNA-stimulated IRF3 signaling. IRF-3 Trans-AM assays were used to measure C10 effects on dsRNA induction of IRF3 DNA binding. Green fluorescent protein-labeled IRF3 was used to measure C10 effects on dsRNA-induced IRF3 nuclear translocation. Native PAGE, SDS PAGE, and western blotting were used to identify effects of C10 on IRF3 homodimer formation and phosphorylation, respectively. There was a significant impairment of dsRNA-induced IRF3 DNA binding activity in human embryonic kidney and pancreatic cancer cells with C10 treatment. C10 also blocked dsRNA-induced IRF3 nuclear translocation and homodimer formation without blocking serine 396 phosphorylation of IRF3. Together, these results indicate that C10 interferes with IRF3 signaling by blocking dsRNA-induced IRF3 homodimer formation, a prerequisite for nuclear translocation and DNA binding activities.

## 1. Introduction

Interferon regulatory factor 3 (IRF3) is a critical mediator of innate immune signaling. IRF3 is activated in response to viral infection or presence of dsRNA in the cytoplasm. In particular, activation of IRF3 by dsRNA, induces serine/threonine phosphorylation of IRF3 near the carboxy-terminus, which leads to the homodimerization of activated IRF3 and translocation of the IRF3 homodimer from the cytoplasm to the nucleus [1,2,3]. In the nucleus the activated IRF3 homodimer complexes with the chaperone proteins CBP and p300, binds to its transcriptional regulatory DNA sequences, and initiates the activation of target gene transcription [*i.e.*, type 1 interferons (*IFNα and IFNβ)*, *RANTES*, *IL-15*, *ISG56*] [1,2,3,4,5,6,7,8,9].

The innate immune response to pathogenic infectious agents is initiated by pattern recognition receptors (PRRs) which recognize pathogen-associated molecular patterns (PAMPs) such as viral dsRNA [10,11,12,13,14]. Currently identified PRRs which recognize cytoplasmic dsRNA and activate IRF3 include toll-like receptor 3 (TLR3), retinoic acid-inducible gene 1 (RIG1), and melanoma differentiation associated factor 5 (MDA5). dsRNA-activated TLR3 interacts with Toll/IL-1 receptor (TIR)-domain-containing adaptor inducing IFN-beta (TRIF), which will subsequently interact with tumor necrosis factor (TNF)R-associated factor 3 (TRAF3) [15]. TRAF3 will then undergo self-ubiquitination to activate TRAF family member-associated NF-kB activator (TANK)-binding kinase 1 (TBK1) and/or inducible IKK (IKK-*i*) [16]. Activated TBK1 or IKK-*i* will then activate IRF3 [10,15,16,17,18,19,20]. The RIG-1-like receptor (RLR) family members, RIG1 and MDA5 undergo conformational changes following binding of dsRNA and expose their caspase recruitment domains (CARDs) [10,21,22]. The CARDs of RIG1 and MDA5 will then interact with the CARD-containing adaptor protein IPS1 (a mitochondrial outer membrane protein also known as MAVS, Cardif, and VISA) that will subsequently activate TRAF3, which in turn activates TBK1 and/or IKK-*i*, which then activates IRF3 [10,17,18,19,20,23,24,25,26,27,28,29]. 

While IRF3 signaling is important for mediating the innate immune response, pathologic IRF3 signaling, via abnormal TLR signaling in non-immune cells for example, can lead to the development of autoimmune and inflammatory diseases such as diabetes (Types 1 and 2), atherosclerosis, autoimmune thyroid disease, inflammatory bowel disease, sepsis, and even cancer [30,31,32,33,34,35,36,37,38,39,40,41,42,43,44,45]. Since autoimmune and inflammatory diseases arising from abnormal TLR signaling afflict millions of individuals worldwide, compounds that inhibit pathologic TLR expression and signaling may be particularly useful as a novel therapeutic approach for these pathologies.

Previous studies have revealed that phenylmethimazole (C10) (structure has been previously described [46]) acts primarily to inhibit dsRNA-triggered IRF3 signaling, preventing dsRNA-induction of type 1 interferon gene expression, type 1 interferon production, and type 1 interferon signaling without cellular toxicity [32,33]. However, the mechanism by which C10 blocks IRF3 transcriptional activity is currently unknown. The studies described herein were designed to explore the molecular basis for C10 inhibition of dsRNA-mediated IRF3 signaling.

## 2. Results and Discussion

### 2.1. C10 Inhibits IRF3 DNA Binding in Human Embryonic Kidney and Pancreatic Cancer Cells

**Figure 1:**
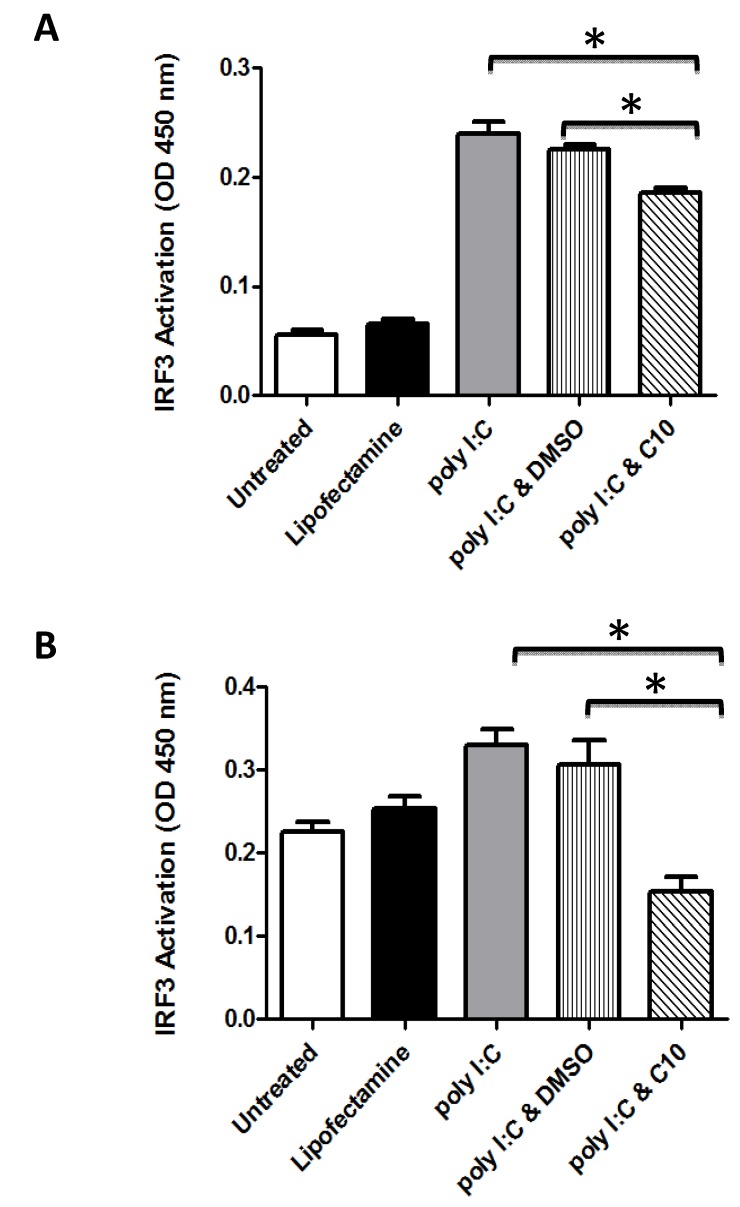
C10 inhibits IRF3 activation in human cell lines. 293-hTLR3-HA (**A**) and PANC-1 (**B**) cells were stimulated with 100 μg/mL Poly I:C during 6 h in the presence or absence of C10 or vehicle (DMSO). IRF3 binding activity in nuclear extracts (5 μg or 10 μg protein respectively) was determined using an ELISA-based technique. Results are presented as mean ± SD from 3 biological replicates. Significance was determined using one-way ANOVA followed by Tukey’s multiple comparison test. * *p* < 0.05 between groups as indicated. All poly I:C treatment groups were statistically different than untreated and lipofectamine control groups, *p* < 0.05.

In order to study the effect of C10 on IRF3 DNA binding activity in human cells, human embryonic kidney 293 (HEK293) cells which stably overexpress human TLR3 tagged with hemagglutinin (HA), HEK293-hTLR3-HA cells, and human pancreatic cancer cells (PANC-1) were transfected with pUNO-hIRF3 as described in the Experimental section. Twenty four h later, cells were incubated with polyinosinic-polycytidylic acid (poly I:C) in the presence or absence of 0.5 mM C10. Six h after stimulation, nuclear proteins were obtained and DNA binding activity was quantified using an ELISA based assay (TransAM transcription factor assay, Active-motif). As shown in Figure 1, C10 treatment produced a significant impairment of the DNA binding activity of IRF3 in both cell types tested which was in agreement with previously reported inhibition of poly I:C-induced IFN transcription in C10 treated cells [33]. 

It is interesting to note that while C10 significantly blocked poly I:C-induced IRF3 activity in both cell types, C10 completely abolished poly I:C-induced IRF3 activity as well as reduced IRF3 activity below basal levels in PANC-1 cells, whereas C10 only partially blocked poly I:C-induced IRF3 activity in 293-hTLR3-HA cells (Figure 1). Observations from Figure 1 may help to explain these differences. First, PANC-1 cells have much higher basal IRF3 activation than 293-hTLR3-HA cells (basal IRF3 activity in PANC-1 cells is roughly twice that of 293-hTLR3-HA cells). Second, poly I:C stimulates IRF3 activity to a much greater extent in 293-hTLR3-HA cells than it does in PANC-1 cells (~4-fold in 293-hTLR3-HA cells, *versus *~two-fold in PANC-1). The higher basal activity of IRF3 in PANC-1 cells (Figure 1) as well as our previous reports that PANC-1 cells basally express much higher levels of IFNβ than HEK293 cells [31], suggests possible chronic activation of endogenous dsRNA-sensing pathways which may be causing the blunted response to exogenous dsRNA (*i.e.*, poly I:C) observed in PANC-1 cells compared to 293-hTLR3-HA cells. In contrast, the lack of basal dsRNA-sensing pathway activity in 293-hTLR3-HA cells as suggested by the observed low basal activity of IRF3 in 293-hTLR3-HA cells, allows for a more dramatic activation of IRF3 activity by exogenous dsRNA (*i.e.*, poly I:C). Since C10 indiscriminately blocks dsRNA activation of IRF3, whether endogenous or exogenous, it makes sense that we would see inhibition of both poly I:C-stimulated IRF3 activity as well as basal IRF3 activity in PANC-1 cells. PANC-1 cells are derived from a human pancreatic carcinoma [47] and thus may contain viral dsRNA or dsRNA of self-origin from cellular damage, which could explain their high basal IRF3 activity. In contrast, 293-hTLR3-HA cells are derived from normal human embryonic kidney cells [48] and are stably transfected with the human TLR3 gene fused to the influenza hemaglutinine (HA) tag. Thus, 293-hTLR3-HA cells over-express human TLR3 at very high levels which may also help explain why they have a more pronounced response to poly I:C. In addition, the partial inhibition of poly I:C-induced IRF3 activation by C10 observed in 293-hTLR3-HA cells may be due to the artificial over-amplification of the TLR3 pathway in these cells.

### 2.2. C10 Inhibits IRF3 Translocation to the Nucleus

**Figure 2:**
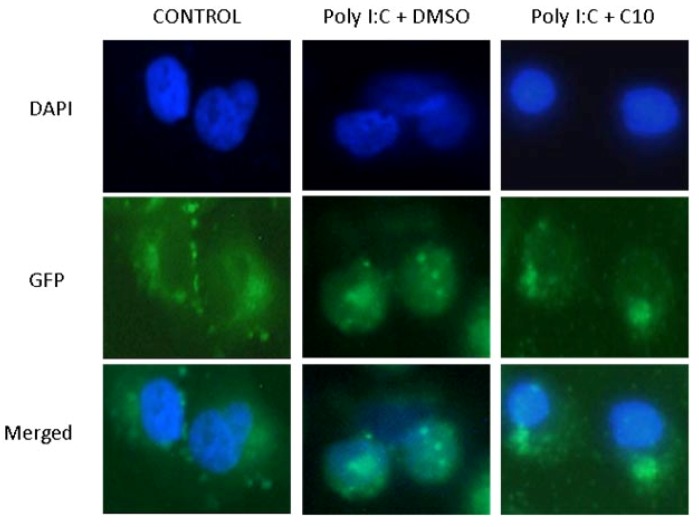
C10 inhibits IRF3 translocation to nucleus. PANC-1 cells were transfected with an expression plasmid for GFP-IRF3. Transfected cells were stimulated with poly I:C in the presence or absence of C10 or vehicle (DMSO). Cells were fixed in 4% formaldehyde and nuclei were stained with DAPI. The sub cellular location of GFP tagged IRF3 was analyzed with an Olympus fluorescence microscope.

In order to establish if C10 was able to block the translocation of activated IRF3 to the nucleus, we used a green fluorescent protein (GFP)-IRF3 fusion protein kindly provided by Dr. Christopher Basler, Mt. Sinai School of Medicine, and which has been used to measure dsRNA-stimulated IRF3 nuclear translocation [49]. Briefly, PANC-1 cells were grown in chamber slides and transfected with 100 ng pEGFP-C1-hIRF3, a plasmid which overexpresses green fluorescent protein (GFP) fused to human IRF3. Twenty-four h later, cells were transfected with poly I:C (1 μg/mL) in the presence or absence of C10, and incubated for 6 h before fixation and counterstaining and examination under a fluorescent microscope. In all cases, proper controls were run (Untreated or Lipofectamine 2000 only). As shown in Figure 2, PANC-1 cells displayed a strong cytoplasmic fluorescence upon transfection with pEGFP-C1-hIRF3. Stimulation of the TLR3 signaling pathway with poly I:C caused the translocation of the fluorescence to the nucleus, indicating translocation of the activated IRF3 homodimer to the nucleus. This translocation was completely blocked by C10 treatment (Figure 2), indicating that C10 blocked translocation of the activated IRF3 homodimer to the nucleus. Similar results were obtained with HEK293-hTLR3-HA cells (data not shown).

### 2.3. C10 Inhibits IRF3 Homodimer Formation

Activation of TLR3 signaling by dsRNA induces serine/threonine phosphorylation of IRF3 near the carboxy-terminus, which leads to the homodimerization of activated IRF3 in the cytoplasm. This event is followed by translocation of the IRF3 homodimer to the nucleus where it will make a complex with specific chaperone proteins and bind to its transcriptional DNA regulatory sequence to initiate transcription of target genes [*i.e.*, type 1 interferons (*IFNα and IFNβ*), *RANTES*, *IL-15*, *ISG56*] [1,2,5,6,7,8].

**Figure 3:**
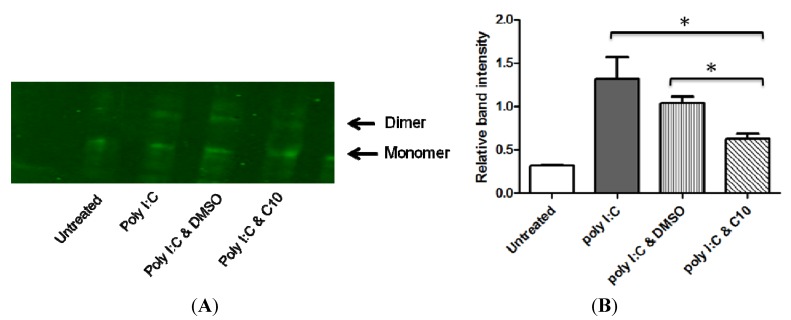
C10 inhibits IRF3 homodimer formation. (**A**) PANC-1 cells were stimulated with poly I:C in the presence or absence of C10 or vehicle (DMSO). Total proteins extracts were separated on a 7.5% nondenaturing polyacrylamide gel with 1% deoxycholate in the cathode buffer. Monomers and dimers were detected by Western Blot analysis using anti-IRF3 antibody. Images shown are representative immunoblots from 3 biological replicates. (**B**) Average relative intensity of dimer to monomer band. Results are presented as mean ± SD from 3 biological replicates. Significance was determined using one-way ANOVA followed by Tukey’s multiple comparison test. * *p *< 0.05 between groups as indicated. All poly I:C treatment groups were significantly different than untreated control group, *p* < 0.05.

If C10 prevents the IRF3 homodimer from entering the nucleus, the compound may be interfering with the translocation itself or with an upstream signaling event. In order to study any upstream effects of C10 on dsRNA-induced IRF3 homodimerization, we performed native polyacrylamide gel electrophoresis (PAGE) followed by Western blotting to analyze proteins isolated from PANC-1 cells that were treated with and without C10. Briefly, PANC-1 cells were transfected with poly I:C (1 μg/mL) and incubated with or without C10 for 6 h. Cellular proteins were collected and separated on a 7.5% non-denaturing polyacrylamide gel with 1% deoxycholate in the cathode buffer as described [50]. IRF3 monomers and dimers were detected by Western Blot analysis using anti-IRF3 antibody. As shown in Figure 3, proteins from resting cells showed only an IRF3 monomer band while the stimulation with poly I:C was associated with an additional homodimer band. Protein extracts obtained from C10-treated cells showed a significant reduction in the intensity of the homodimer band. These results suggest that C10 treatment interferes with dsRNA-stimulated IRF3 homodimer formation.

### 2.4. C10 Does Not Affect IRF-3 Phosphorylation

**Figure 4:**
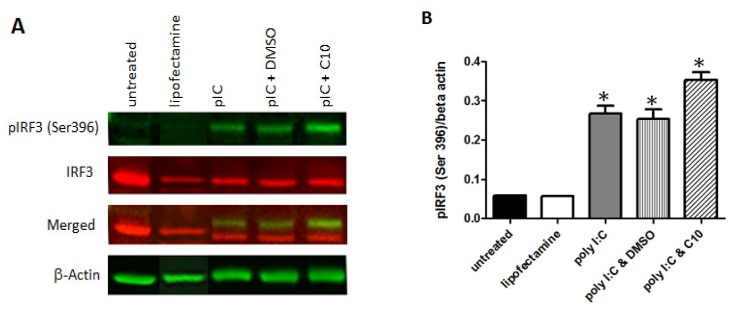
C10 does not inhibit IRF3 phosphorylation of Ser 396. (**A**) PANC-1 cells were stimulated with poly I:C in the presence or absence of C10 or vehicle (DMSO). Total protein extracts were separated using denaturing SDS PAGE. Total IRF3 and Ser 396 phosphorylated IRF3 were detected by Western Blot analysis using anti-IRF3 and anti-phospho-Ser 396 IRF3 antibodies. Images shown are representative immunoblots from 3 biological replicates. (**B**) Average relative intensity of phospho-IRF3 to beta actin after Odyssey scanning. Results are presented as mean ± SD from 3 biological replicates. Significance was determined using one-way ANOVA followed by Tukey’s multiple comparison test. * Indicates significant difference from untreated and lipofectamine control groups as indicated, *p* < 0.002. There was no significant difference between poly I:C, poly I:C & DMSO, and poly I:C & C10 groups.

IRF3 is post-translationally modified by phosphorylation at multiple serine and threonine residues located in the carboxy terminus of IRF3. Point mutations of residues Ser396 and Ser398 eliminated virus-induced phosphorylation and nuclear translocation of IRF3 protein [51], establishing their importance in this pathway. Given that IRF3 phosphorylation is a necessary event that precedes the formation of the IRF3 homodimer, we subsequently evaluated effects of C10 on dsRNA-stimulated IRF3 phosphorylation. To accomplish this, we activated TLR3 signaling using poly I:C transfection in PANC-1 and HEK293-hTLR3-HA in the presence or absence of C10 and proper controls. Total proteins were analyzed by Western blotting to evaluate total or serine 396 phosphorylated IRF3. As shown in Figure 4, C10 did not block dsRNA-induced phosphorylation of IRF3 at serine residue 396 in any of the cell types tested. Taking into account that IRF3 has multiple phosphorylation sites [51], this negative result does not rule out that phosphorylation in other residues can be affected by C10.

It is noteworthy to mention that no cellular toxicity was observed in 293-hTLR3-HA or PANC-1 cells treated with C10 in these studies, an observation in agreement with our previous report using C10 at the same concentration in PANC-1 and HEK293 cells [31]. Data presented in Figure 2 indicate that cellular morphology of C10-treated cells is identical to control cells, confirming the absence of cellular toxicity. In addition, data in Figure 4 show that the internal control (beta actin) protein expression is unaffected by C10 treatment also indicative of the absence of cellular toxicity associated with C10 treatment. 

## 3. Experimental

### 3.1. Phenylmethimazole (C10) Solutions

Phenylmethimazole was provided by the Interthyr Corporation. C10 was prepared as a fresh 200 mM stock solution in 100% (v/v) DMSO, and further diluted into medium to achieve the concentrations indicated in individual experiments.

### 3.2. Cell Culture

All experiments were performed with 293-hTLR3-HA (InvivoGen) and PANC-1 cells, generously provided by Dr. Duxin Sun (The Ohio State University). 293-hTLR3-HA cells were cultured in DMEM (Invitrogen) containing 10% (v/v) fetal bovine serum (ATLAS) and 10 μg/mL blasticidin (InvivoGen). PANC 1 cells were grown in the same basal media containing 10% (v/v) fetal bovine serum (ATLAS), 100 U/mL penicillin and 100 μg/mL streptomycin (Invitrogen). Both cell lines were grown at 37 °C with 5% CO_2_. 

### 3.3. Plasmids and Transfections

pEGFP-IRF3 plasmid was kindly provided by Dr. Christopher Basler (Mount Sinai School of Medicine) and was used for IRF3 nuclear translocation assays [49]. For IRF3 phosphorylation experiments, pUNO-hIRF3-HA plasmid (InvivoGen) was used. All transfections were performed using Lipofectamine 2000 (Invitrogen) with standard protocol provided by the manufacturer.

### 3.4. Preparation of Nuclear Extracts for IRF-3 DNA-Binding Activity

293-hTLR3-HA and PANC-1 were used to study IRF3 DNA-binding activity. For these purposes, cells were grown to 70% confluence in 10 mm^2^ dishes and transfected with 100 ng pUNO-hIRF3 per dish. Twenty four h after transfection, cells were stimulated with 100 μg/mL poly I:C in the presence or absence of 0.5 mM C10. Six h after stimulation nuclear proteins were obtained using a Nuclear Extraction Kit (Active Motif) and protein concentration was quantified using the Micro BCA Protein Assay kit (Thermo Scientific).

### 3.5. Detection of IRF-3 DNA-Binding Activity

IRF3 binding activity in nuclear extracts was measured with an ELISA based assay (Trans-AM IRF3 transcription, Active Motif) according to the manufacturer’s protocol. Nuclear extracts (5 μg for 293-hTLR3-HA cells or 10 μg for PANC-1) were incubated on plates coated with ISRE consensus oligonucleotide. Following incubation and extensive washes, the quantity of bound transcription factor was then determined by use of a specific primary antibody and a secondary antibody conjugated to horseradish peroxidase in an ELISA-based format. The intensity of the reaction was measured at 450 nm.

### 3.6. Translocation of IRF-3 to the Nucleus

In order to further assess the mechanism by which C10 prevented IRF3 transcriptional activity, we proposed to determine whether C10 had an effect on translocation of activated IRF3 to the nucleus following poly I:C activation of the TLR3 signaling pathways using a GFP-IRF3 fusion as previously reported [49]. To accomplish this, 293-hTLR3-HA and PANC-1 cells were grown in 8 well chamber slides to 70–80% confluence. Cells were then transfected with 100 ng pEGFP-hIRF3, a plasmid which overexpresses green fluorescent protein (GFP) fused to human IRF3. Twenty-four h later, cells were transfected with poly I:C (1 μg/mL), and incubated for 6 h in the presence or absence of C10 or DMSO. In all cases, proper controls were run (Untreated or Lipofectamine 2000 only). Cell monolayers were rinsed with cold PBS, fixed for 5 minutes with 4% formalin and counter stained with 4',6-diamidino-2-phenylindole, dihydrochloride (DAPI) for nuclear localization of the IRF3 signal and then examined under fluorescent microscopy. 

### 3.7. SDS and Native PAGE Analyses

PANC-1 cells were grown on 10 cm dishes to confluence. Cells were transfected or not with poly I:C (1 ug/mL) to induce IRF3 phosphorylation in the presence or absence of C10. Six h later, whole cell extracts were isolated. Briefly, cells were washed in ice-cold phosphate-buffered saline before being lysed on ice in buffer (10 mM Tris-HCl pH 7.5,150 mM NaCl, 1% Nonidet P-40 (v/v) and protease inhibitor cocktail (set III EDTA free, Calbiochem). The cell lysates were centrifuged at 13,000 rpm for 15 min. Protein concentration in supernatants (whole cell extracts, WCE) was determined using micro BCA Protein Assay kit (Thermo Scientific) according to manufacturer’s instructions.

For SDS-PAGE, 10 μg of protein extracts per lane were electrophoresed on 4–12% acrylamide gradient bis-Tris gels (Invitrogen). Native PAGE electrophoresis in order to study IRF3 dimer formation was performed as described previously [50]. Briefly, 7.5% acrylamide gels without SDS (Bio-Rad) were pre-run with 25 mM Tris and 192 mM glycine, pH 8.4, with and without 1% deoxycholate (Sigma) in the cathode and anode chamber, respectively, for 30 min, at 40 mA. WCEs (10 μg) diluted in the native sample buffer (62.5 mM Tris-HCl, pH 6.8, 15% glycerol, and bromophenol blue) were applied to the gel and subjected to electrophoresis for 60 min at 25 mA. 

Proteins were transferred to nitrocellulose membranes, blocked with LI-COR blocking buffer and incubated with primary antibody (anti-total IRF3 and anti-Phospho Ser 396 IRF3, Cell Signaling) followed by appropriate secondary antibody. All antibody dilutions were performed in LI-COR blocking buffer supplemented with 0.2% Tween 20 (Calbiochem). Membranes were scanned using the Odyssey Infrared Imaging System (LI-COR)

### 3.8. Statistical Analysis

All experiments were replicated at least three times on different groups of cells. All data were expressed as mean ± SD. One-way ANOVA followed by Tukey’s multiple comparison test were used to evaluate statistical significance of the results with GraphPad Instat. 

## 4. Conclusions

The results described herein indicate that phenylmethimazole (C10) interferes with dsRNA-induced IRF3 signaling specifically by (**1**) preventing DNA binding activity of IRF3; (**2**) blocking dsRNA-induced IRF3 translocation to the nucleus; and (**3**) impairing IRF3 homodimer formation, a prerequisite for nuclear translocation. C10 does not, however, affect phosphorylation of IRF3 at serine residue 396 in PANC-1. Future work will focus on determining whether or not C10 binds IRF3 directly to prevent homodimerization, or if C10 blocks IRF3 homodimer formation by blocking an essential phosphorylation and/or other post-translational modification necessary for IRF3 homodimer formation.
